# Association Study of Tumor Necrosis Factor Receptor 1 (*TNFR1*) Gene Polymorphisms with Schizophrenia in the Polish Population

**DOI:** 10.1155/2017/6016023

**Published:** 2017-11-29

**Authors:** Renata Suchanek-Raif, Krzysztof Kucia, Małgorzata Kowalczyk, Paweł Raif, Monika Paul-Samojedny, Anna Fila-Daniłow, Jan Kowalski

**Affiliations:** ^1^School of Pharmacy with the Division of Laboratory Medicine in Sosnowiec, Medical University of Silesia, Katowice, Poland; ^2^Department of Medical Genetics, Medical University of Silesia, Jedności 8 Street, 41-200 Sosnowiec, Poland; ^3^School of Medicine in Katowice, Medical University of Silesia, Katowice, Poland; ^4^Department of Psychiatry and Psychotherapy, Medical University of Silesia, Ziołowa 45 Street, 40-635 Katowice, Poland; ^5^Department of Biosensors and Biomedical Signals Processing, Faculty of Biomedical Engineering, Silesian University of Technology, Roosevelta 40 Street, Zabrze, Poland

## Abstract

Schizophrenia is a devastating mental disorder with undetermined aetiology. Previous research has suggested that dysregulation of proinflammatory cytokines and their receptors plays a role in developing schizophrenia. We examined the association of the three single nucleotide polymorphisms (SNPs; rs4149576, rs4149577, and rs1860545) in the tumor necrosis factor receptor 1 *(TNFR1)* gene with the development and psychopathology of paranoid schizophrenia in the Polish Caucasian sample consisting of 388 patients and 657 control subjects. The psychopathology was assessed using a five-factor model of the Positive and Negative Syndrome Scale (PANSS). SNPs were genotyped using the TaqMan 5′-exonuclease allelic discrimination assay. The SNPs tested were not associated with a predisposition to paranoid schizophrenia in either the entire sample or after stratification according to gender. However, rs4149577 and rs1860545 SNPs were associated with the intensity of the PANSS excitement symptoms in men, which may contribute to the risk of violent behavior. Polymorphisms in the *TNFR1* gene may have an impact on the symptomatology of schizophrenia in men.

## 1. Introduction

Schizophrenia is a complex mental disorder that is characterized by abnormalities of thought, emotion, and behavior. Its etiology still remains elusive. The possible role of the immune response system in the pathogenesis of schizophrenia is indicated by the observation of abnormalities in the levels of many cytokines in the serum and cerebrospinal fluid (CSF), imbalances in the type-1 and type-2 immune responses, and the increased production of antibodies [1].

Several lines of research have suggested that the dysregulation of the inflammatory response plays a role in schizophrenia. A wide range of data has shown that the levels of the proinflammatory cytokine interleukin-1*β* (IL-1*β*), IL-6, IL-12, tumor necrosis factor-*α*, TNF-*α*, and interferon-*γ* (INF-*γ*) and their soluble receptors are increased in both the serum and CSF compared to healthy people [1, 2]. A postmortem study reported that inflammation-related genes are overexpressed [3]. An increased activation of microglia and astrocytes in schizophrenia has also been revealed. The neural activities of the proinflammatory cytokines are primarily mediated by microglia [4]. Cytokines play a pleiotropic role in the central nervous system, which includes their influence on synaptic plasticity, neurotransmission, or neurogenesis [2, 4–6]. Data from a large genome-wide association (GWA) study revealed new genes that play roles in immunity are also connected with the risk of developing schizophrenia [7].

TNF-*α* is a strong candidate gene for schizophrenia susceptibility. An elevated level of TNF-*α* has been found in patients with schizophrenia [6]. Many results have shown multiple function TNF-*α* in CNS. This proinflammatory cytokine has some activities that can affect both brain development and the adult brain. TNF-*α* that is derived from a peripheral immune cell can also cross the blood-brain barrier [8]. TNF-*α* shows its activity through two receptors—tumor necrosis factor receptor 1 (TNFR1; also known as p55 or TNFRSF1A) and TNFR2 (p75, TNFRSF1B). Both receptors are constitutively expressed by all neural cell types [8].

TNFR1 plays a pivotal role by inducing various cellular responses including cell death, differentiation, or inflammation [9]. Animal models with targeted deficiencies in the *TNFR1* gene showed many CNS abnormalities such as lesions and death of neurons especially in the hippocampus, which is also damaged in schizophrenia [9]. In the course of schizophrenia, the level of TNFR1 is increased in both the brain [10] and serum [5, 11, 12]. An association between the level of TNFR1 and the severity of the psychotic symptoms of schizophrenia has also been found [11–13]. The TNFR1 biological pathway was associated with the risk of developing schizophrenia in a GWA study [14].

The TNFR1 receptor exists in two forms—one that is membrane-bound and one that is soluble (sTNFR1) in serum. Both forms are encoded by the same gene. The soluble forms are released from the transmembrane forms of the receptors by the proteolytic processing that is carried by the TNF-*α* converting enzyme (TACE) [15]. Many papers have shown that the serum level of the soluble form sTNFR1 is significantly increased in the course of schizophrenia [11, 12]. The membrane-bound receptors of TNFR1 and TNFR2 were also increased in the lymphocytes of schizophrenia patients [5].

Altered levels of both the soluble and membrane-bound forms of TNFR1 have been found in both the serum and the lymphocytes in other psychiatric disorders such as bipolar disorder [11, 16] or depression [17, 18]. An association between the level of TNFR1 and the intensity of the symptoms of those diseases has also been shown. In individuals suffering from depression, an elevated expression of the *TNFR1* gene was negatively correlated with cognitive efficiency [17]. In schizophrenia and bipolar disorder, high plasma levels of TNFR1 have been associated with more severe psychotic symptoms [11]. Evidence from genetics and symptomatology research has implied that there are connections between schizophrenia and bipolar disorder. More than one hundred genes are shared between these syndromes [19, 20]. The sTNFR1 was negatively correlated with global functioning in schizophrenia. Moreover, they showed that both patients with treatment-resistant schizophrenia and with concomitant depression had an increased level of sTNFR1 than other patients [12].

The aim of the present work was to evaluate the potential association between three TNFR1 single nucleotide polymorphisms (SNPs) (rs4149576, rs4149577, and rs1860545) and schizophrenia in a Caucasian population. All of the patients suffered from the paranoid subtype of schizophrenia. Firstly, we evaluated the distribution of genotypes, alleles, and haplotypes in a case-control study. Secondly, we assessed whether these SNPs were associated with the psychotic symptomatology of paranoid schizophrenia, as well as any family history of schizophrenia or suicide attempts. To the best of our knowledge, this is the first work that investigates the association between *TNFR1* gene polymorphisms and schizophrenia in Caucasian individuals.

## 2. Methods

### 2.1. Subjects

The study subjects (*n* = 1045) consisted of 388 patients and 657 healthy controls. The study was approved by the Bioethics Committee of the Medical University of Silesia. All of the participants were of Caucasian Polish origin and lived in the Silesia region. They were assessed to be capable of understanding the study and provided written consent before inclusion, and their anonymity was preserved. Inclusion criteria were a diagnosis of the paranoid subtype of schizophrenia. Exclusion criteria were the presence of depressive episodes and comorbid mental disorders including anxiety disorder, schizoaffective disorder, and organic brain disease or substance dependence. In addition, endocrine and autoimmune diseases constituted the exclusion criteria.

The patients were recruited from inpatients being treated at the Clinic of Psychiatry in Katowice and the Neuropsychiatric Hospital in Lubliniec. The patients were diagnosed with schizophrenia according to the *Diagnostic and Statistical Manual of Mental Disorders, 4th Edition, Text Revision* (DSM-IV-TR) criteria. The degree of the severity of the psychotic symptoms was evaluated by two independent psychiatrists using the Positive and Negative Scale (PANSS).The results of PANSS were presented in a five-factor model encompassing factors of positive (POS), negative (NEG), disorganization (DIS), excitement (EXC), and emotional distress (EMO) symptoms. The numerical values of these factors were calculated according to the formula given by the author, using all 30 items of PANSS [21]. The age of onset was defined as the first appearance of positive psychotic symptoms.

The 74 patients had attempted suicide. In all cases, suicide attempts were confirmed by family members or significant others as well as by medical records. A suicide attempt is a self-inflicted, potentially injurious behavior with a nonfatal outcome for which there is evidence of intent to die. Parasuicidal patients were not included into the group of patients with suicide attempts. The data on family burden were obtained from interview with family as well as by medical records.

The control group had been recruited from among volunteer blood donors. Exclusion criteria for controls were current psychiatric problems, any other neurological disorders, and a family history of schizophrenia (verified during the interview) and chronic and acute physical illness such as an infection, autoimmune, or allergic diseases.

### 2.2. Genotyping

Genomic DNA was isolated from peripheral blood using QIAampDNA Blood Mini Kit (Qiagen, Valencia, CA). The SNPs were genotyped using the TaqMan 5′-exonuclease allelic discrimination assay; the assay ID (Thermo Fisher Scientific Inc) of each SNP was C_2645705_10 for rs4149576; C_2645708_10 for rs4149577; C_12029112_10 for rs1860545. Thermal cycling conditions for polymerase chain reaction (PCR) were 1 cycle at 95°C for 10 min followed by 40 cycles of 95°C for 15 s (denaturation) and 60°C for 1 min (annealing/extension).

### 2.3. SNP Choice

The *TNFR1* gene is located on the 12p13.31 chromosome. We selected SNPs according to the following criteria: (1) SNPs should have a high minor allele frequency (MAF ≥ 20%) in order to maximize power (information retrieved from a public database, the National Center for Biotechnology Information; dbSNP, http://www.ncbi.nlm.nih.gov/SNP/); (2) SNPs should be located in the intron of the gene; and (3) SNPs should have previously been associated with neurological disorders or others complex human phenotypes.

### 2.4. Statistical Analysis

Descriptive variables are presented as mean ± standard deviation (SD), as well as median (Me) and range (minimum-maximum). Qualitative data are expressed as percentage values. Differences in the allele, genotype, and haplotype frequencies between control and patient groups were compared by the *χ*^2^ test. The two-way ANOVA and Tukey's HSD (honest significance test) post hoc test was used for comparisons of PANSS subscales and the age of onset between patients with genotypes. Homogeneity of variance was assessed by the Levene test. The extent of linkage disequilibrium (LD) expressed in terms of D′ and *r*^2^ coefficients, haplotype frequencies, and possible departure from the Hardy-Weinberg equilibrium (HWE) was estimated using the SNPStats software. Statistical calculations were performed by using the STATISTICA version 10 (http://www.statsoft.com) and SNPStats (bioinfo.inconcologia.net) and RStudio. All results with *p* < 0.05 were accepted as statistically significant.

## 3. Results

### 3.1. Case-Control Study

#### 3.1.1. Sample Characteristics

One thousand forty-five (*n* = 1045) subjects were enrolled in this study. Among the 388 patients, 151 (39%) were women and 237 (61%) were men; the mean age ± standard deviation (SD) was 41.1 ± 12.4 years. The mean ± SD of the PANSS scores for the positive, negative, disorganization, emotional distress, and excitement subscales were 23.1 ± 5.5, 23.3 ± 5.9, 31.7 ± 6.9, 22.1 ± 4.9, and 20.4 ± 5.6, respectively. The mean age of the onset of the disease was 25.4 ± 6.8 years. The control group consisted of 657 unrelated subjects and included 315 (48%) women and 342 (52%) men. The mean age ± SD was 40.8 ± 8.6 years.

#### 3.1.2. Power Analysis

The power of the test of statistical significance is the probability that the test will reject a false null hypothesis. Statistical power depends on (1) significance level (alpha), (2) effect size parameter, and (3) the size of the samples.

The significance level (alpha) was 0.05. We have used effect size at level 0.2 which is considered small according to Cohen [22]. In our research, the smallest sample size was for the G/G genotype for the rs4149577 SNP. For this reason, the rs4149577 with a minimum ratio of G/G (17%) was selected for power analysis. The result showed that this number of samples (65) gives sufficient power (>0.97). This was the smallest power value among all that we computed. In our computations, we used the R statistical software.

#### 3.1.3. Distribution of Genotypes and Alleles

The allele distribution did not significantly depart from the Hardy–Weinberg Equilibrium (HWE) neither among patients with schizophrenia (rs4149576, *p* = 0.76; rs4149577, *p* = 0.30; rs1860545, *p* = 0.92) nor for controls (rs4149576, *p* = 0.64; rs4149577, *p* = 0.18; rs1860545, *p* = 0.94).  Table 1 shows the frequencies of the genotypes and alleles of the three SNPs of the *TNFR1* gene among the patients and the controls. There were no statistically significant differences in the distribution of genotypes and alleles between the patients and the control group for any of the SNPs that were analysed. Also, after stratification according to gender, the distribution of both genotypes and alleles did not statistically significantly differ between patients and controls neither among men nor women (Table 1).

#### 3.1.4. Linkage Disequilibrium and Haplotype Analysis

All three SNPs were in the strong linkage disequilibrium (LD). LD for rs4149576 and rs4149577 was *D*′ = 0.97, *r*^2^ = 0.87; *p* < 0.0001, for rs4149576 and rs1860545 was *D*′ = 0.99, *r*^2^ = −0.94; *p* < 0.0001, and for rs4149577 and rs1860545 was D′ = 0.96, *r*^2^ = −0.82; *p* < 0.0001. The haplotype distribution did not significantly differ between patients and controls.

### 3.2. Case Study

#### 3.2.1. Suicide Attempts

Among the 388 patients, 74 (19%) had attempted suicide; mean age ± SD 38 ± 10.9 years (median (me) = 37, range 21–65 years). The mean age ± SD of disease onset was 24.4 ± 5.7 years (me = 23, range 17–43 years). There were 47 (64%) men (mean age ± SD 36.8 ± 10.4 years, me = 36, range 22–60) and 27 (36%) women (mean age ± SD 41.4 ± 11.4 years, me = 41, range 21–65 years) in this group. There were no statistically significant differences in the distribution of the genotypes, alleles (Table 2), or haplotypes among the patients who had or had not attempted suicide for either the total sample or when stratified according to gender (data not shown, *p* > 0.05).

#### 3.2.2. Family History of Schizophrenia

There were 95 patients with a family history of schizophrenia; mean age ± SD 41.3 ± 12.1 years (me = 41, range 18–70 years). The mean age ± SD of onset was 22.2 ± 6.1 years (me = 24, range 13–24 years). There were 60 (63%) men (mean age ± SD: 37.3 ± 11.6 years, me = 47, range 18–60) and 35 (37%) women (mean age ± SD: 48.0 ± 10.0 years, me = 47, range 31–70 years) in this group. There were no statistically significant differences in neither the distribution of genotypes and alleles (Table 2) nor haplotypes (data not shown, *p* > 0.05) in the total sample or when stratified according to gender between patients with and without a family history of schizophrenia.

#### 3.2.3. Distribution of Genotypes and Alleles among the Men and Women with Schizophrenia

The group of men and women with schizophrenia did not show any significant differences in the distribution of either the genotypes or alleles for any of the SNPs that were analysed (Table 3).

#### 3.2.4. Age of Onset and PANSS Subscales

**Figure 1:**
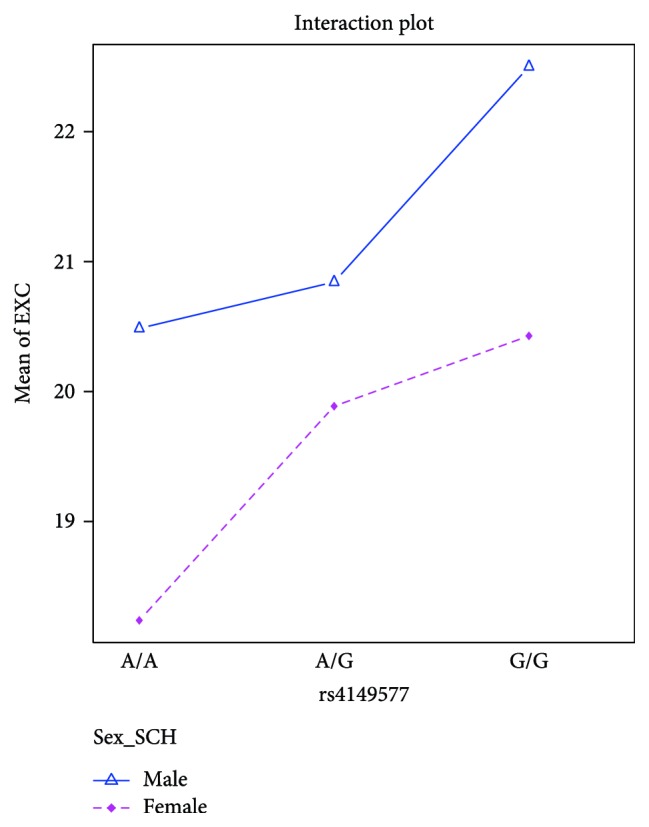
The results of the ANOVA analysis for rs4149577 and EXC subscale in the patient group.

We performed the ANOVA test in order to examine the impact of the genotypes of rs4149576, rs4149577, and rs1860545SNPs on the psychopathology, which was measured using the five-factor model of the PANSS scale as well as the age of the onset of paranoid schizophrenia. We found statistical differences for rs4149577 in the EXC scale. A patient with the rare G/G genotype (less common) had higher scores in the EXC scale than patients with the A/A genotype (*F* = 7.02; df = 1, *p* < 0.01). This was especially true for men with the G/G genotype who had more intense EXC symptoms compared to the women with the A/A genotype (*p* < 0.01) (Figure 1).

**Figure 2:**
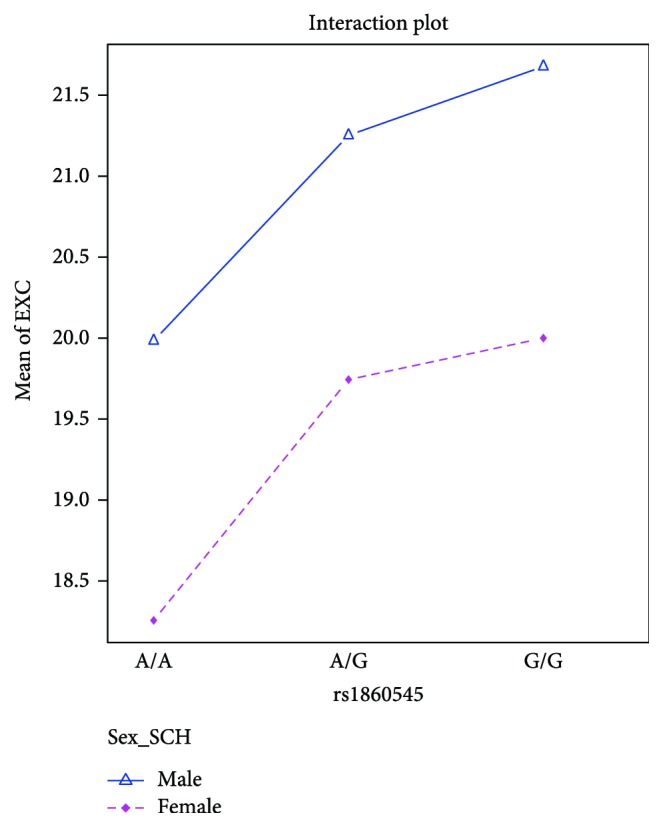
The results of the ANOVA analysis for rs1860545 and EXC subscale in the patient group.

We also found a tendency of statistical differences for rs1860545 in the EXC scale. A patient with the G/G genotype, which was frequent, had more points in the EXC scale than patients with the less common A/A genotype (*F* = 7.58; df = 1, *p* = 0.06). This was especially true for the men with the G/G genotype who had more intense EXC symptoms than women with the A/A genotype (*p* < 0.01) (Figure 2).

There were no statistical differences between the POS, NEG, DIS, and EMO scales and any of the SNPs that were analysed (*p* > 0.05, data not shown). We also did not find any statistical differences between the age of the onset of paranoid schizophrenia and the genotypes of the tested SNPs (*p* > 0.05, data not shown).

## 4. Discussion

The identification of genes is one of the aspects that is necessary to understand an individual's susceptibility to schizophrenia. Much data has suggested that the dysregulation of proinflammatory cytokines and their receptors plays a key role in developing schizophrenia [4, 23, 24]. In this paper, we focused on three SNPs (rs4149576, rs4149577, and rs1860545) in the *TNFR1* gene, which encodes the major receptor for the TNF-*α* cytokine. The SNPs that were tested have been implicated with complex human phenotypes in previous papers. Both rs4149576 and rs4149577 SNPs have been associated with the volume of striatal grey matter in healthy individuals [25] and inflammatory demyelinating disease as well [26]. The rs1860545 and rs4149576 SNPs have also been associated with multiple sclerosis [27, 28].

Our case-control study suggests that these SNPs are not associated with paranoid schizophrenia. We did not observe any differences in the distribution of the genotypes and alleles in either the entire group or the group that was stratified according to gender. To the best of our knowledge, this is the first study that investigated the association of *TNFR1* gene single nucleotide polymorphisms with schizophrenia.

In the analysis of the association between the *TNFR1* genotypes and the course of schizophrenia, we found an association between the rs4149577 and PANSS excitement scale (EXC). Patients with the less common genotype (G/G) had more intense excitement symptoms than patients with the A/A genotype. This was especially true when men with the G/G genotype were compared to women with the A/A genotype. We obtained similar results, for rs1860545. The patients with the G/G genotype had higher scores in the EXC scale than patients with the less common genotype (A/A), especially and obviously when men with the G/G genotype were contrasted to women with the A/A genotype. However, in this case, the G/G genotype is the frequent (more common) genotype of rs1860545. No changes in the age at the onset of schizophrenia were observed that were dependent on the SNPs that were tested. To the best of our knowledge, this is the first study that investigated the association between the rs4149576, rs4149577, and rs1860545 polymorphic sites of the *TNFR1* gene and the symptom severity of paranoid schizophrenia.

The excitement cluster (excitement, hostility, uncooperativeness, tension, and impulsivity) may contribute to the risk of violent behavior, nonadherence to treatment, and the likelihood of discharge and substance use in psychosis [29, 30]. Recent research has revealed an association between a reduced caudate nucleus volume and both rs4149576 G/G and rs4149576 C/C homozygotes in healthy subjects [25]. Those results are consistent with our results, which also show an association of SNP rs4149577, especially the less common allele (G/G in our study) with excitement symptoms, which may be linked to the striatum. The caudate nucleus is one of the structures that comprise the dorsal striatum, which is a component of the basal ganglia. The role of the basal ganglia in the emotional and cognitive processes has been proven [31]. Dysfunctions of some of the basal ganglia circuits are associated with emotional changes such as mania, anxiety, and depression. A dysfunction of the lateral orbitofrontal circuit causes personality changes, which are marked by a loss of control or inflexible behavior. The striatum is likely to be involved in the reward processes and motivated behavior [32].

Single nucleotide polymorphisms have been analysed in many cytokines, in particular, those that are associated with altered levels in schizophrenia such as TNF-*α*, IL1-*β*, IL-6, IL-10, INF-*γ*, and in the genes encoding the immune system regulatory proteins CTLA-4 and CD28 [33–37]. SNPs have been identified in promoters, coding regions, introns, and 3′-flanking regions of cytokine genes. The legitimacy of studies of the SNPs in those genes is supported by the results from a GWA study. The risk of developing schizophrenia is strongly connected with the genes that participate in either the inflammation process or the function of the immune system [7]. A GWA study that examined genes that were arranged by their biological pathways found a strong association for the transforming growth factor *β* (TGF-*β*) signaling pathway and the TNFR1 pathway [14].

Individuals with schizophrenia have a higher suicide risk than those in the general population. The suicide risk is estimated to be 4-5% and is especially high during the first year after the diagnosis [38]. There is growing evidence that the cytokine network may play a role in the pathophysiology of suicidal behavior. It has been found that the IL-2, IL-6, IL-8, and TNF-*α* levels are altered for both subjects with suicidal ideation and suicide attempters. Several authors have shown a higher plasma level of TNF-*α* in victims of suicide [24, 39–43]. Our study showed that the SNPs of the *TNFR1* gene that were tested are not involved in suicide attempts in individuals with paranoid schizophrenia. We also did not observe any characteristic haplotypes for those individuals. However, this is possibly due to the small number of subjects.

Evidence from family studies suggests that there is a higher risk for developing schizophrenia among relatives of an individual with this disorder. The greatest risk is among first-degree relatives. Heritability was estimated to be up to 70–80% [44]. The family transmission patterns have suggested that a family's risk of schizophrenia is higher among the relatives of schizophrenic women than those of schizophrenic men [45]. We also did not find any associations between the SNPs that were analysed and a family history of schizophrenia. To the best of our knowledge, this type of analysis was conducted in a Caucasian population for the first time.

## 5. Conclusions

The focus of this study was to evaluate the association between the three SNPs of the *TNFR1* gene. This study was conducted on a homogeneous group of patients with the paranoid subtype of schizophrenia who had no coexisting depressive episodes or schizoaffective disorders. Our data showed that those SNPs are not associated with a predisposition to develop paranoid schizophrenia in either men or women. However, the SNPs rs4149577 and rs1860545 may play a role in the intensity of the excitement symptoms of paranoid schizophrenia in men. The excitement symptoms may contribute to the risk of violent behavior, nonadherence to treatment, and the likelihood of discharge and substance use in psychosis.

## 6. Limitations

The study was limited geographically (Polish population). For this reason, it may not be universally applicable. Further studies with larger samples size are needed to validate our results.

## Figures and Tables

**Table 1:** Genotype and allele distributions for the *TNFR1* (rs4149576, rs4149577, and rs1860545) polymorphisms in paranoid schizophrenia patients (*n* = 388) and control subjects (*n* = 657).

Polymorphisms	*N* (%)								
	Genotype					Allele			
rs4149576	T/T	C/T	C/C	*χ* ^2^	*p*	T	C	*χ* ^2^	*p*
Patients	108 (28)	190 (49)	90 (23)	*0.34*	*0.84*	406 (52)	370 (48)	*0.35*	*0.55*
Control	174 (26)	322 (49)	161 (25)			670 (51)	644 (49)		
Male patients	64 (27)	114 (48)	59 (25)	*0.68*	*0.71*	242 (51)	232 (49)	*0.34*	*0.55*
Male controls	82 (24)	172 (50)	88 (26)			336 (49)	348 (51)		
Female patients	44 (29)	76 (50)	31 (21)	*0.47*	*0.78*	164 (54)	138 (46)	*0.08*	*0.76*
Female controls	92 (29)	150 (48)	73 (23)			334 (53)	296 (47)		
rs4149577	A/A	A/G	G/G	*χ* ^2^	*p*	A	G	*χ* ^2^	*p*
Patients	122 (31)	201 (52)	65 (17)	*3.07*	*0.21*	445 (57)	331 (43)	*0.02*	*0.88*
Control	222 (33)	305 (46)	130 (19)			749 (57)	565 (43)		
Male patients	72 (30)	121 (51)	44 (19)	*0.40*	*0.81*	265 (56)	209 (44)	*0.04*	*0.83*
Male controls	105 (31)	167 (49)	70 (20)			377 (55)	307 (45)		
Female patients	50 (33)	80 (53)	21 (14)	*3.8*	*0.14*	180 (60)	122 (40)	*0.03*	*0.88*
Female controls	117 (37)	138 (44)	60 (19)			372 (59)	258 (41)		
rs1860545	G/G	G/A	A/A	*χ* ^2^	*p*	A	G	*χ* ^2^	*p*
Patients	96 (25)	193 (50)	99 (26)	*0.79*	*0.67*	391 (50)	385 (50)	*0.69*	*0.41*
Control	174 (26)	330 (51)	153 (23)			636 (48)	678 (52)		
Male patients	62 (26)	115 (49)	60 (25)	*0.91*	*0.63*	235 (50)	239 (50)	*0.60*	*0.43*
Male controls	95 (28)	172 (50)	75 (22)			322 (47)	362 (53)		
Female patients	34 (23)	78 (52)	39 (26)	*0.36*	*0.83*	156 (52)	146 (48)	*0.20*	*0.65*
Female controls	79 (25)	158 (50)	78 (25)			314 (50)	316 (50)		

**Table 2:** Genotype and allele distributions for the *TNFR1* (rs4149576, rs4149577, and rs1860545) SNPs in patients with and without family history of schizophrenia and suicide attempts.

Polymorphisms	*N* (%)								
	Genotype					Allele			
rs4149576	T/T	C/T	C/C	*χ* ^2^	*p*	T	C	*χ* ^2^	*p*
With family history	24 (25)	48 (51)	23 (24)	*0.41*	*0.81*	96 (49)	94 (51)	*0.23*	*0.67*
Without family history	84 (29)	142 (48)	67 (23)			276 (47)	310 (53)		
With suicide attempts	17 (23)	41 (55)	16 (22)	*1.64*	*0.43*	75 (51)	73 (49)	*0.12*	*0.72*
Without suicide attempts	91 (29)	149 (47)	74 (24)			331 (53)	297 (47)		
rs4149577	A/A	A/G	G/G	*χ* ^2^	*p*	A	G	*χ* ^2^	*p*
With family history	31 (33)	48 (50)	16 (17)	*0.09*	*0.95*	110 (58)	80 (42)	*0.01*	*0.92*
Without family history	91 (31)	153 (52)	49 (17)			335 (57)	251 (43)		
With suicide attempts	23 (31)	40 (54)	11 (15)	*0.28*	*0.86*	86 (58)	62 (42)	*0.01*	*0.90*
Without suicide attempts	99 (32)	161 (51)	54 (17)			359 (57)	269 (43)		
rs1860545	G/G	G/A	A/A	*χ* ^2^	*p*	A	G	*χ* ^2^	*p*
With family history	26 (27)	46 (49)	23 (24)	*0.47*	*0.78*	92 (48)	98 (52)	*0.29*	*0.58*
Without family history	70 (24)	147 (50)	76 (26)			299 (51)	287 (49)		
With suicide attempts	17 (23)	41 (55)	16 (22)	*1.24*	*0.53*	73 (49)	75 (51)	*0.03*	*0.84*
Without suicide attempts	79 (25)	152 (48)	83 (27)			318 (51)	310 (49)		

**Table 3:** Genotype and allele distributions for the *TNFR1* (rs4149576, rs4149577, and rs1860545) SNPs in women and men with paranoid schizophrenia.

Polymorphisms	*N* (%)								
	Genotype					Allele			
rs4149576	T/T	C/T	C/C	*χ* ^2^	*p*	T	C	*χ* ^2^	*p*
Women with schizophrenia	44 (29)	76 (50)	31 (21)	*1.0*	*0.6*	164 (54)	138 (46)	*0.65*	*0.41*
Men with schizophrenia	64 (27)	114 (48)	59 (25)			242 (51)	232 (49)		
rs4149577	A/A	A/G	G/G	*χ* ^2^	*p*	A	G	*χ* ^2^	*p*
Women with schizophrenia	50 (33)	80 (53)	21 (14)	*1.47*	*0.47*	180 (60)	122 (40)	*0.88*	*0.37*
Men with schizophrenia	72 (30)	121 (51)	44 (19)			265 (56)	209 (44)		
rs1860545	G/G	G/A	A/A	*χ* ^2^	*p*	A	G	*χ* ^2^	*p*
Women with schizophrenia	34 (22)	78 (52)	39 (26)	*0.68*	*0.71*	156 (52)	146 (48)	*0.24*	*0.62*
Men with schizophrenia	62 (26)	115 (49)	60 (25)			235 (50)	239 (50)		
